# Selective Recognition of Myoglobin in Biological Samples Using Molecularly Imprinted Polymer-Based Affinity Traps

**DOI:** 10.1155/2018/4359892

**Published:** 2018-08-08

**Authors:** Rüstem Keçili

**Affiliations:** Anadolu University, Yunus Emre Vocational School of Health Services, Department of Medical Services and Techniques, 26470 Eskisehir, Turkey

## Abstract

The current work demonstrates the design, characterization, and preparation of molecularly imprinted microspheres for the selective detection of myoglobin in serum samples. The suspension polymerization approach was applied for the preparation of myoglobin imprinted microspheres. For this purpose, N-methacryloylamino folic acid-Nd^3+^ (MAFol- Nd^3+^) was chosen as the complex functional monomer. The optimization studies were performed changing the medium pH, temperature, and myoglobin concentration. pH 7.0 was determined as the optimum value where the prepared imprinted microspheres displayed maximum binding for myoglobin. The maximum binding capacity was achieved as 623 mgg^−1^. In addition, the selectivity studies were conducted. The results confirmed that the imprinted microspheres showed great selectivity towards myoglobin in the existence of hemoglobin, cytochrome c, and lysozyme which were chosen as potentially competing proteins.

## 1. Introduction

Myoglobin (Mb) is a hemeprotein which is primarily found in muscle tissues which binds oxygen through its heme functional group. Mb is also a crucial biomarker for the early recognition of various diseases such as acute myocardial infarction also called “heart attack” [1–3]. Because of its small size, Mb in muscle tissues is released to the bloodstream when muscle tissue is damaged. The increase in the level of Mb may lead to kidney failure because it may be converted to the undesired toxic molecules [4, 5]. After the acute myocardial infarction, Mb level in serum starts to increase in a short time and its level reaches the maximum value in 6-9 h. Therefore, the early determination of Mb level is vital for the detection of acute myocardial infarction [6–13].

Several conventional approaches such as enzyme-linked immunosorbent assay (ELISA) [14, 15], chromatography [16–19], electrochemical sensors [20–24], and surface plasmon resonance (SPR) sensors [25–27] were applied for the detection of myoglobin. Most of these approaches show high sensitivity towards myoglobin. However, they have some disadvantages such as high production costs (especially for antibody-based assays), high process time, low stability, and need of special and expensive equipment. Thus, innovative approaches that have higher selectivity are required.

Molecularly imprinted polymers (MIPs) also known as “artificial antibodies” are artificial materials that possess specific binding regions towards a desired molecule. During the preparation of selective MIPs, appropriate functional monomers are chosen and polymerized with a cross-linker in the presence of the desired molecule (template). Since MIPs display excellent affinity and selectivity towards the target compound, they can efficiently be used in different applications such as biosensor platforms, catalysis, and extraction [28–30]. In addition, MIPs are low-cost and robust materials at harsh process conditions such as high pressure, high temperature, and low and high pH values [31–37].

In our previous study [38], we developed a molecularly imprinted cryogel column using the functional monomer N-methacryloylamino antipyrine for the recognition of myoglobin. Unlike this reported study, molecularly imprinted microspheres which show excellent selectivity and binding capacity towards myoglobin in serum samples compared to other reported studies were prepared in the present work. For this purpose, the complex functional monomer N-methacryloylamino folic acid-Nd^3+^ (MAFol-Nd^3+^) was polymerized with the target protein myoglobin (template) in the presence of ethylene glycol dimethacrylate which is the cross-linker. To the best of our knowledge, this is the first report in which the complex functional monomer MAFol-Nd^3+^ was used for the design and preparation of selective imprinted microspheres towards proteins. The prepared myoglobin imprinted microspheres were characterized and their binding behaviour towards target protein myoglobin was evaluated.

## 2. Experimental Section

### 2.1. Chemicals

Myoglobin, hemoglobin, lysozyme, cytochrome c, folic acid, 2,2′-azobisisobutyronitrile, poly (vinyl alcohol) (molecular weight: 27.000), ethylene glycol dimethacrylate, and solvents were provided by Sigma-Aldrich (St. Louis, MO, USA).

### 2.2. Analytical Instruments

FT-IR spectroscopic studies were conducted using Perkin-Elmer 400 FT-IR spectrometer. The morphological characterization of the imprinted microspheres was done using scanning electron microscope (SEM) (FEI-Quanta-FEG 250 model) and Shimadzu-UV-3600 model spectrophotometer was used for the UV spectroscopic studies. Circular dichroism (CD) spectroscopic studies of the extracted and commercial myoglobin were carried out by using Applied Photophysics Chirascan CD Spectropolarimeter.

### 2.3. The Synthesis of the Functional Monomer MAFol

The MAFol was successfully synthesized by applying the following recipe which has been already reported [39].

Firstly, folic acid (1 eq) in H_2_O was prepared and solution pH was then adjusted to 9.0 with 1 M NaOH. Then, 15 mL of methacryloyl benzotriazole in dioxane was mixed with this solution. The final mixture was allowed to stir for 60 min. Once the reaction was finished, evaporation of dioxane was carried out and excess of the benzotriazole was removed by extraction using EtOAc. Then, the pH of the aqueous phase was adjusted to pH 6. Finally, the product MAFol was obtained after aqueous phase was removed from the reaction medium.

**Figure 1:**
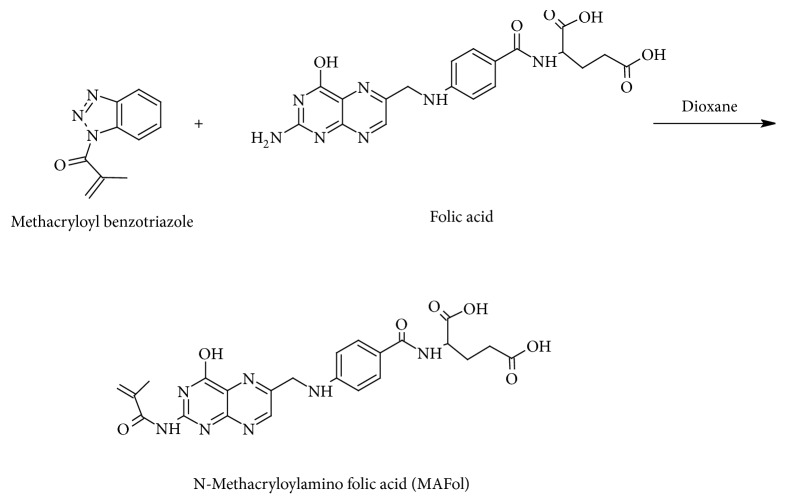
MAFol synthesis.

The MAFol synthesis is schematically demonstrated in Figure 1.

### 2.4. Preparation of Complex Monomer MAFol- Nd^3+^

For the preparation of MAFol- Nd^3+^, 1.0 mmol Nd (NO_3_)_3_.6H_2_O was added to 2.0 mmol MAFol solution in chloroform and stirred for 18 h. After filtration, the obtained complex was washed with H_2_O and EtOH and allowed to dry at 55°C for 18 h.

### 2.5. Preparation of Imprinted Microspheres for Myoglobin

Suspension polymerization was used for the preparation of the myoglobin imprinted microspheres (MIPs). The following protocol was applied.

0.2 g poly (vinyl alcohol) in 50 mL H_2_O was prepared for the dispersion phase. Then, 100 mg MAFol-Nd^3+^-myoglobin preorganized complex monomer in 5 mL DMSO was added to the mixture of ethylene glycol dimethacrylate: toluene (1.0 mL:5.0 mL). The prepared final solution was then mixed with the aqueous dispersion phase. Then, initiator 2,2′-azobisisobutyronitrile (ca. 30 mg) was added and the solution was stirred at 70°C for 8 h and 90°C for the next 4 h. Once the polymerization was completed, the prepared myoglobin imprinted microspheres (MIPs) were washed with EtOH and deionized H_2_O and dried at 55°C for 18 h. Template removal from the imprinted microspheres was carried out by extraction with 1.0 M NaCl for 24 h. The same procedure was used for the preparation of nonimprinted microspheres (NIPs) without myoglobin.

### 2.6. Characterization Studies

SEM, FT-IR analyses, and swelling tests were performed for the characterization experiments of the prepared MIPs and NIPs. To perform the FT-IR studies, 10 mg of the dry particles was placed on the ATR crystal surface and the FT-IR spectrum was recorded.

For the SEM analyses, thin gold layer (approximately 20 nm) was deposited on the surface of the microspheres to provide conductivity. The SEM images were then recorded.

The swelling tests of the prepared MIPs and NIPs were also carried out. In these experiments, the dried MIPs were put into the distilled water in a NMR tube for 3 h. Then, the volume of the MIPs in swollen state was determined.

Equation (1) was used for the calculation of the % swelling ratio of the prepared polymers.

(1) $$\begin{array}{c} \%\text{ Swelling ratio}=\left[\;\frac{V\;swollen-V\;dry}{Vdry}\;\right]\times 100 \end{array}$$

where*** Vdry*** is volume of the MIPs in dry state and***Vswollen*** is volume of the MIPs in swollen state.

For the CD spectroscopic measurements, 500 ppm myoglobin in pH 7.0 phosphate buffer was prepared. Analyses of the samples were carried out using a quartz cuvette. The CD spectra were obtained at 180-340 nm wavelength.

### 2.7. Binding of Myoglobin to the Imprinted Microspheres

In the binding studies for myoglobin in batch mode, 20 mg of each MIP and NIP was put into glass vials. Then, 2 mL of 0.5 mgmL^−1^ myoglobin in pH 7.0 phosphate buffer was added and these mixtures were allowed to stir for 1 h and then aliquots of 1 mL were taken and analyzed by UV-VIS spectrophotometer at 410 nm wavelength.

To evaluate the pH effect on the binding of myoglobin to the MIP/NIP, 2 mL of 0.5 mgmL^−1^ myoglobin in different buffer solutions pH 4 to 9 was added to 20 mg of the polymer. Then, the solutions were allowed to stir for 1 h and 1 mL aliquots of each solution were spectrophotometrically analyzed at 410 nm.

On the other hand, 2 mL of 0.5 mgmL^−1^ myoglobin in pH 7.0 phosphate buffer was added to 20 mg of MIP and NIP to test the time effect on the binding of myoglobin. The solutions were stirred and aliquots of 1 mL of each solution were taken at different time (10, 20, 30, 40, 50, and 60 min) and spectrophotometrically analyzed at 410 nm.

The effect of myoglobin concentration on binding efficiency of the polymer was also studied. 2 mL solution of various concentrations of myoglobin in pH 7.0 phosphate buffer was interacted with 20 mg of MIP and NIP. The solutions were then allowed to stir for 1 h and 1 mL aliquots of each solution were spectrophotometrically analyzed at 410 nm.

### 2.8. Reusability of the Imprinted Microspheres and Selectivity Studies for Myoglobin

To evaluate the reusability of the imprinted microspheres towards myoglobin, myoglobin binding cycles were repeated 10 times using the same material. Then, microspheres were washed with 1.0 M NaCl after each cycle for the regeneration.

The studies for the selectivity of the prepared imprinted microspheres for myoglobin were conducted in the presence of hemoglobin, cytochrome c, and lysozyme which were chosen as competitor proteins. For this purpose, 20 mg imprinted microspheres were put into the 2 mL of 0.5 mgmL^−1^ solution composed of myoglobin, hemoglobin, cytochrome c, and lysozyme in pH 7.0 phosphate buffer. The samples were allowed to stir for 1 h. Then, 1 mL of the collected aliquots was spectrophotometrically analyzed at 410 nm.

### 2.9. Binding of Myoglobin in Human Serum

In these experiments, 20 mg of MIP and NIP was put into glass vials and 0.1 *μ*gmL^−1^ myoglobin was spiked in the 2 mL of serum sample and interacted with 20 mg of MIP/NIP. Then, the solutions were allowed to stir for 1 h. 1 mL of the solutions was then spectrophotometrically analyzed at 410 nm.

## 3. Results and Discussion

### 3.1. Characterization Experiments of the Imprinted Microspheres towards Myoglobin

**Figure 2:**
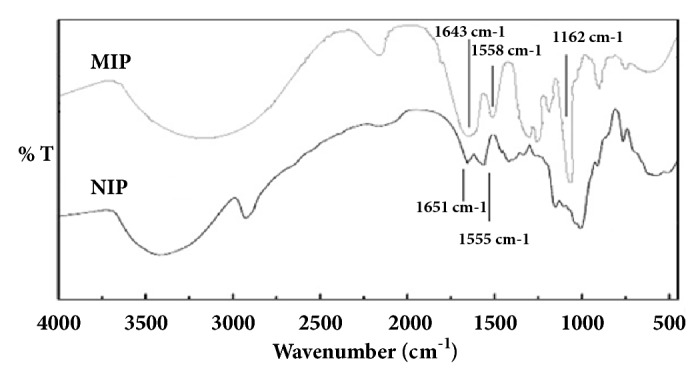
FT-IR spectra of imprinted microspheres (MIP) and nonimprinted microspheres (NIP).

The results obtained from the FT-IR experiments are shown in Figure 2. As seen, the obtained FT-IR spectra of MIP and NIP in Figure 2 were similar which confirms that the prepared MIPs and NIPs have similar backbone. In the FT-IR spectra, absorption peaks observed at 1645 cm^−1^ (COO- stretching) and at 2900 cm^−1^ (C-H stretching) belong to the functional monomer and the peak at 1160 cm^−1^ (C-O stretching) belongs to the cross-linker.

**Figure 3:**
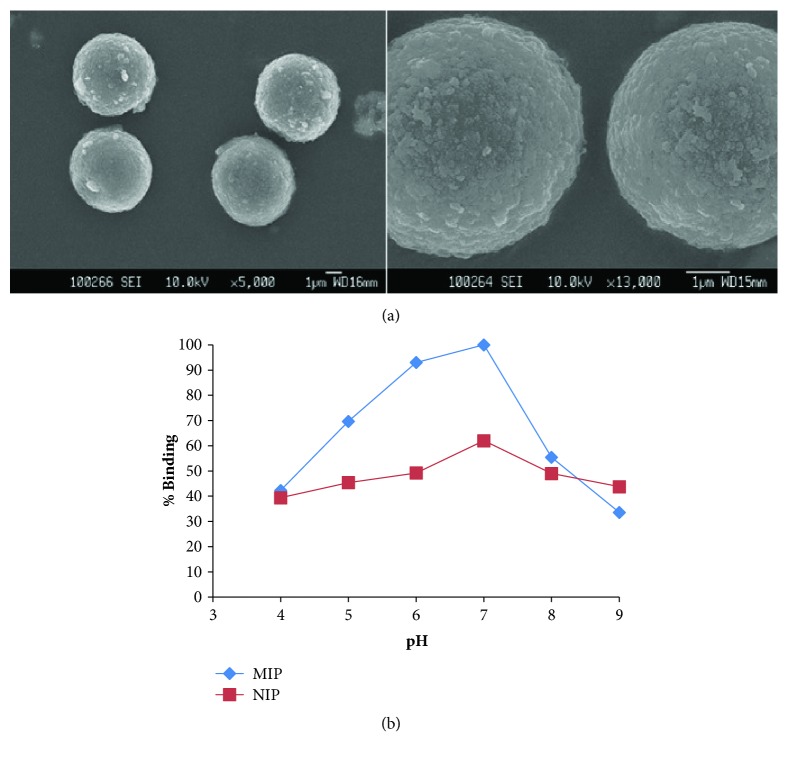
(a) SEM images of th**e **myoglobin imprinted microspheres. (b) The pH effect on myoglobin binding [C_Mb_: 0,5 mgmL^−1^, m_polymer_: 20 mg, t: 1 h].

The obtained SEM images of the myoglobin imprinted microspheres are shown in Figure 3(a). The images indicated that the prepared imprinted microspheres are porous and spherical. The swelling behaviours of the microspheres in H_2_O were also investigated. The swelling ratios of the MIP and NIP were obtained as 57.4% and 36.3%, respectively.

### 3.2. pH, Time, Ionic Strength, and Concentration Effects on the Binding of Myoglobin to the Imprinted Microspheres

The pH effect on the binding of myoglobin was evaluated in the pH range between 4.0 and 9.0. The obtained experimental outcomes are shown in Figure 3(b). The outcomes confirmed that the maximum binding of myoglobin was achieved at pH 7.0.

The reason of this result can be explained by the strong coordination and electrostatic interaction of myoglobin and the complex functional monomer MAFol-Nd^3+^ through Nd^3+^ ions at pH 7.0. Ionization states of various groups on the amino acid residues of the myoglobin may also lead to high binding of myoglobin at this pH value. The binding behaviour of the myoglobin imprinted microspheres considerably decreased at higher and lower than pH 7.0. The repulsive electrostatic forces between Nd^3+^ ions and myoglobin cause the decrease in the myoglobin binding

**Figure 4:**
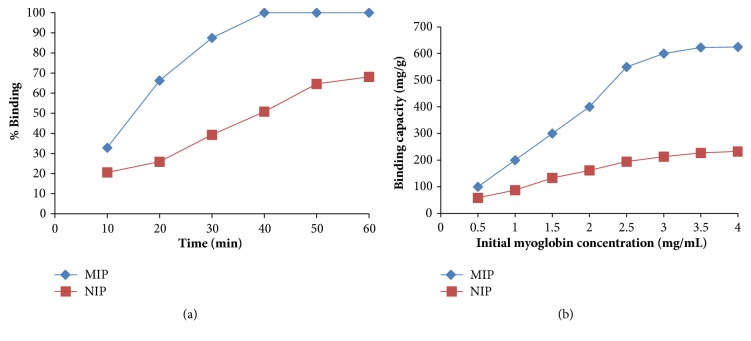
(a) The time effect on the binding of myoglobin [C_Mb_:0,5 mgmL^−1^, m_microbeads_: 20 mg, pH: 7.0, t: 1 h]. (b) Effect of initial myoglobin concentration on binding [m_microbeads_: 20 mg, pH: 6.0, t: 1 h, T: 25°C].

The binding kinetics were also carried out to evaluate how the binding capability of the imprinted microspheres for myoglobin changes over time. For this purpose, the experiments were conducted using 0.5 mgmL^−1^ myoglobin. The obtained results are shown in Figure 4(a). The binding of myoglobin to the imprinted microspheres gradually increased within 40 min. Then, the myoglobin binding reached an equilibrium. Myoglobin binding to the imprinted microspheres is fast at the beginning of binding process. Then, it becomes much more difficult due to the difficulties on the penetration of myoglobin molecules into the MIP particles. The rapid binding of myoglobin until 40 min can be explained by the strong affinity interactions and complexation between the complex functional monomer MAFol-Nd^3^ and the target protein myoglobin.

The myoglobin concentration effect on the binding is shown in Figure 4(b). As seen, the imprinted microspheres displayed high binding behaviour towards myoglobin at higher myoglobin concentrations in the range from 0.5 to 3.5 mgmL^−1^. After saturation value which is 3.5 mgmL^−1^, the myoglobin binding to the imprinted microbeads reached an equilibrium since myoglobin molecules occupied all binding regions of the imprinted microspheres. The highest binding of myoglobin to the imprinted microspheres was achieved as 623 mgg^−1^.

Ertürk and her colleagues developed cryogel-based column systems for the detection of myoglobin in human plasma. In their study, N-methacryloyl-(L)-tryptophan (MATrp) was chosen as the functional monomer [40]. The highest binding myoglobin was obtained as 35.9 6 mgg^−1^.

Turan et al. prepared imprinted hydrogels towards myoglobin using N-isopropylacrylamide and 2-acrylamido-2-methyl-propanosulfonic acid. The maximum myoglobin binding was determined as 97.40 mgg^−1^ [41].

**Figure 5:**
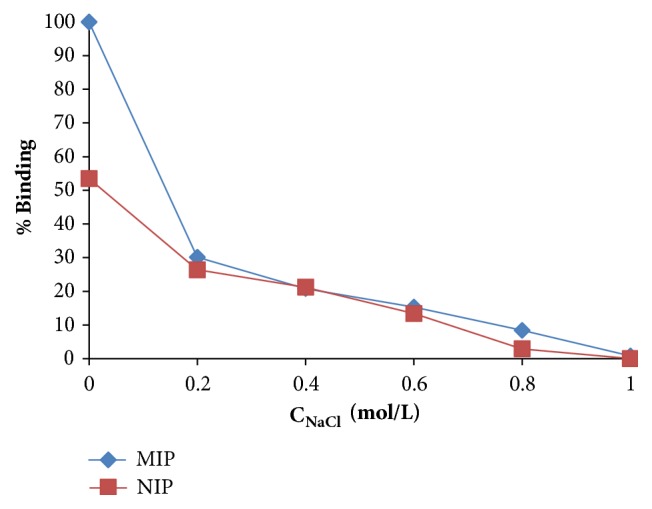
The ionic strength effect on myoglobin binding [m_microbeads_: 20 mg, pH: 7.0, t: 1 h, T: 25°C].

The ionic strength effect on the binding of myoglobin was also investigated (Figure 5). The binding behaviour of the imprinted microspheres for myoglobin significantly decreased at higher concentrations of NaCl that was changed from 0 to 1.0 M. The reason of this can be the ionic interactions of the counter NaCI ions with the myoglobin molecules. This ionic interaction may lead to masking of the binding regions of the microspheres towards myoglobin. In addition, the repulsive electrostatic interactions between the microspheres and myoglobin molecules at higher NaCl concentrations may also cause the decrease of myoglobin binding to polymer. The nonspecific binding behaviour of nonimprinted microspheres towards myoglobin can be explained by the cooperative effects of various binding mechanisms such as ion-exchange or hydrophobic interactions.

### 3.3. Binding Isotherms

Binding isotherms were used for the characterization of the interactions between myoglobin and the prepared imprinted microspheres.

In the Langmuir binding isotherm [42], binding data can be obtained using the following:

(2) $$\begin{array}{c} \frac{1}{Q}=\left[\;\frac{1}{Qmax\times b}\;\right]\left[\;\frac{1}{Ceq}\;\right]+\frac{1}{Qmax} \end{array}$$

In this equation,***Q*** represent the myoglobin amount bound to the MIP (mgg^−1^),***Qmax*** is the highest myoglobin binding to the imprinted microspheres (mgg^−1^),***Ceq*** is the myoglobin concentration at equilibrium (mgL^−1^), and***b*** is the Langmuir constant that shows the affinity interactions between myoglobin and imprinted microspheres.

Another binding isotherm is Freundlich isotherm [43], which is defined in the following:

(3) $$\begin{array}{c} \log Q=\log kF+\frac{1}{n}\log Ceq \end{array}$$

where***Ceq*** is the concentration of myoglobin at equilibrium (mgL^−1^),***kF*** is the Freundlich constant, and **n** is the Freundlich exponent.** 1/n** is a measure of heterogeneity of the myoglobin binding regions of the imprinted microspheres changes from 0 to 1. When** 1/n** value gets closer to zero heterogeneity increases.

The obtained results from the equations above for myoglobin binding to the polymers are shown in Table 1. The obtained results confirmed that the Langmuir binding isotherm well demonstrates the myoglobin binding to the prepared imprinted microspheres. The determined Langmuir constant** b** for myoglobin was 5.34 mLmg^−1^.

### 3.4. Reusability of the Imprinted Microspheres and Selectivity Studies for Myoglobin

**Figure 6:**
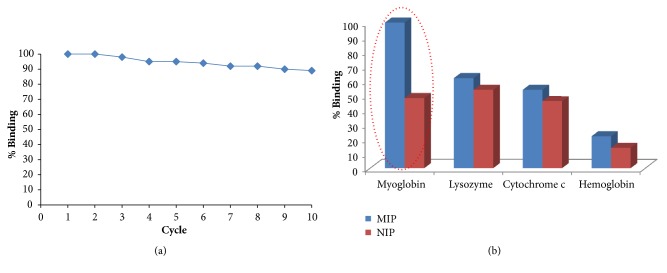
(a) Reusability of the myoglobin imprinted microbeads [C_Mb_: 0,5 mgmL^−1^, m_microbeads_: 20 mg, pH: 7.0, t: 1 h, T: 25°C]. (b) Selectivity of the MIPs and NIPs for myoglobin.

In this part of the study, same microspheres were used 10 times after each binding cycle. The obtained outcomes are shown in Figure 6(a). The prepared imprinted microspheres for myoglobin preserved their robustness even after 10 cycles.

The selectivity of the imprinted microspheres towards myoglobin was also tested. For this purpose, cytochrome c, lysozyme, and hemoglobin were chosen as competitive proteins. The imprinted microspheres towards myoglobin displayed excellent selectivity for myoglobin in the existence of hemoglobin, cytochrome c, and lysozyme (Figure 6(b)). On the other hand, nonimprinted microspheres showed lower binding behaviour for myoglobin. Although their isoelectric points (pIs) are very close to each other (pI of myoglobin is 6.9 and pI of hemoglobin is 7.2), the molecular weights of myoglobin and hemoglobin are 17 and 65 kDa, respectively. In addition, the shapes of these two proteins are completely different. Myoglobin has ellipsoidal shape while the hemoglobin has biconcave shape. Because the 3D cavities in the polymeric network of the imprinted microspheres are matched to the shape of the myoglobin, the entrance and binding of different molecules having different shapes and molecular weights to the 3D cavities are difficult.

### 3.5. Myoglobin Extraction from Real Samples

**Figure 7:**
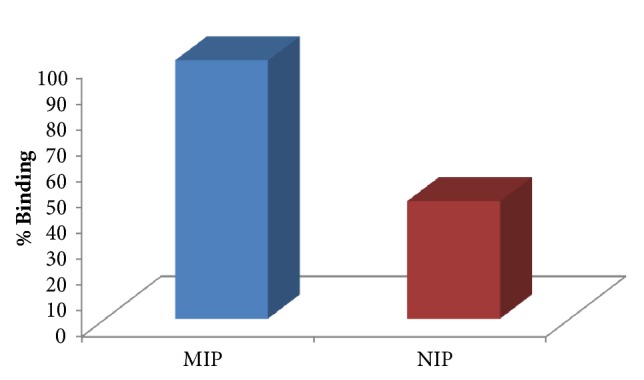
Extraction of myoglobin from human serum [C_Mb_: 0.1 *μ*gmL^−1^, m_polymer_: 20 mg, pH: 7.0, t: 1 h, T: 25°C].

The results from myoglobin extraction from human serum are given in Figure 7. The imprinted microspheres (MIP) showed 100% binding towards myoglobin while the nonimprinted microspheres (NIP) exhibited 59% binding.

**Figure 8:**
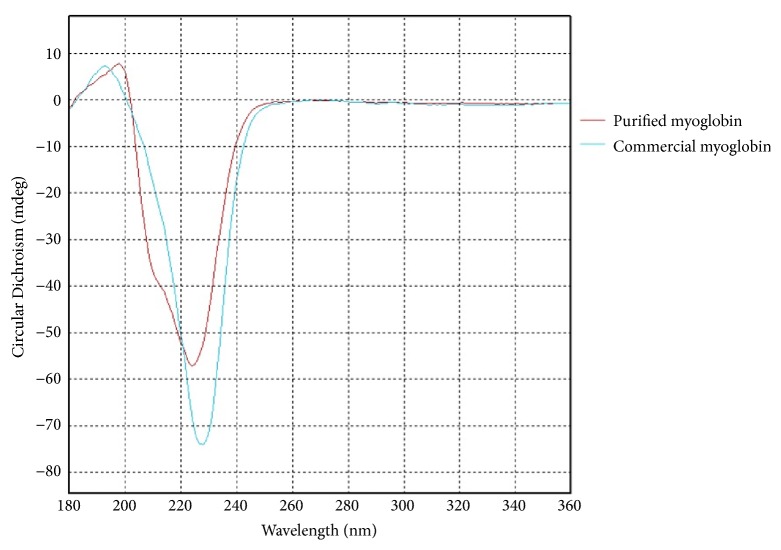
CD spectra of purified myoglobin and commercial myoglobin.

CD analyses of the commercial and extracted myoglobin from human serum were also performed to obtain detailed information about the secondary structure of the myoglobin backbone.  Figure 8 shows the CD spectra of commercial and extracted myoglobin from human serum. As can be seen from the figure, bands between 190 and 230 nm show the *α*-helical type of secondary structure of 500 mg/mL myoglobin (blue spectrum) [44, 45]. The red spectrum showed that *α*-helical type of secondary structure in 208-222 nm interval remained for isolated myoglobin. Peaks between 208 and 222 nm interval showed this data. This means that the isolated protein has preserved the secondary structure without any deformation in its structure during isolation processes.

## 4. Conclusions 

In the current study, molecular imprinting-based microspheres were prepared for the selective detection and extraction of myoglobin from human serum. The outcomes confirmed that the prepared imprinted microspheres can be efficiently employed for the selective detection of target protein myoglobin in the existence of various competing proteins such as hemoglobin, cytochrome c, and lysozyme. The achieved maximum binding capacity was 623 mgg^−1^ at pH 7.0

In conclusion, the current study demonstrated the design and development of low-cost, reusable, robust, and selective materials using molecular imprinting technique for the sensitive recognition of a crucial protein myoglobin in real samples.

## Figures and Tables

**Table 1:** The data calculated from the Freundlich and Langmuir isotherm equations.

**Experimental**	**Langmuir**			**Freundlich**		
**Q (mgg** ^**-1**^ **)**	**Q (mgg** ^**-1**^ **)**	**b (mLmg** ^**-1**^ **)**	**R** ^**2**^	**K**_**F**_ **(mgg** ^**-1**^ **)**	**n**	**R** ^**2**^
623	615.15	5.34	0.98	583.45	3.35	0.93

## Data Availability

All relevant data are within the article and are available from the corresponding author upon request.
